# The Vpu-interacting Protein SGTA Regulates Expression of a Non-glycosylated Tetherin Species

**DOI:** 10.1038/srep24934

**Published:** 2016-04-22

**Authors:** Abdul A. Waheed, Scott MacDonald, Maisha Khan, Megan Mounts, Maya Swiderski, Yue Xu, Yihong Ye, Eric O. Freed

**Affiliations:** 1 Virus-Cell Interaction Section, HIV Dynamics and Replication Program, Center for Cancer Research, National Cancer Institute, Frederick, MD 21702-1201, USA; 2 Laboratory of Molecular Biology, NIDDK, Bethesda, MD 20892, USA

## Abstract

The HIV-1 accessory protein Vpu enhances virus release by counteracting the host restriction factor tetherin. To further understand the role of host cell proteins in Vpu function, we carried out yeast two-hybrid screening and identified a previously reported Vpu-interacting host factor, small glutamine-rich tetratricopeptide repeat-containing protein (SGTA). While RNAi-mediated depletion of SGTA did not significantly affect levels of tetherin or virus release efficiency, we observed that overexpression of SGTA inhibited HIV-1 release in a Vpu- and tetherin-independent manner. Overexpression of SGTA in the presence of Vpu, but not in its absence, resulted in a marked stabilization and cytosolic relocalization of a 23-kDa, non-glycosylated tetherin species. Coimmunoprecipitation studies indicated that non-glycosylated tetherin is stabilized through the formation of a ternary SGTA/Vpu/tetherin complex. This accumulation of non-glycosylated tetherin is due to inhibition of its degradation, independent of the ER-associated degradation (ERAD) pathway. Because the SGTA-stabilized tetherin species is partially localized to the cytosol, we propose that overexpression of SGTA in the presence of Vpu blocks the translocation of tetherin across the ER membrane, resulting in cytosolic accumulation of a non-glycosylated tetherin species. Although our results do not provide support for a physiological function of SGTA in HIV-1 replication, they demonstrate that SGTA overexpression regulates tetherin expression and stability, thus providing insights into the function of SGTA in ER translocation and protein degradation.

Human immunodeficiency virus type 1 (HIV-1), the causative agent of AIDS, encodes three essential structural polyproteins (Gag, Pol and Env), two regulatory proteins (Tat and Rev), and four accessory proteins (Vif, Vpr, Vpu and Nef)^1^. The *vpu* gene is present in HIV-1 and certain simian immunodeficiency viruses (SIVs; SIVgsn, SIVmus, SIVmon) but not in HIV-2 or other SIVs. Vpu is a 16-kDa, type I integral membrane phosphoprotein that is expressed from a bicistronic mRNA together with the Env glycoprotein^2^. Vpu contains an amino-terminal transmembrane (TM) domain and a carboxy-terminal cytoplasmic tail (CT). The CT of Vpu consists of two α-helices linked by a short loop. Two serine residues (S52 and S56) that undergo phosphorylation to recruit β-TrCP, a key component of the SkpI-Cullin-F-box E3 ubiquitin ligase complex, are located in the short loop^3^. Vpu is primarily localized in the ER and Golgi and also to some extent at the plasma membrane^4^.

The two primary functions of Vpu are (i) degradation of CD4, the primary receptor for HIV-1 and other primate lentiviruses^5^,^6^,^7^ and (ii) enhancement of the release of newly formed virus particles from the cell surface by inhibiting the activity of the host restriction factor tetherin/BST-2/CD317/HM1.24 (hereafter referred to as tetherin)^8^,^9^. The degradation of CD4 involves the interaction of Vpu and CD4 via their cytoplasmic domains, followed by recruitment of β-TrCP to the Vpu-CD4 complex, which leads to ubiquitylation and proteasomal degradation of CD4^10^. In this case, Vpu acts as a linker between CD4 and β-TrCP. In contrast, enhancement of virus release involves the TM domain of Vpu to counteract the antiviral activity of tetherin^11^. Vpu also downregulates the expression of major histocompatibility complex class II^12^ and tetraspanin proteins^13^,^14^ from the cell surface. It has been reported that Vpu also protects HIV-1-infected cells from antibody-dependent cell-mediated cytotoxicity (ADCC) through down-regulation of CD4 and tetherin^15^.

Tetherin is an interferon-inducible protein that inhibits virus release by trapping mature virions on the cell surface^8^,^9^. It is an ~180 amino acid, type-II integral membrane protein that contains a short, N-terminal CT domain, a TM domain, a rod-like coil-coil ectodomain, and a glycosylphosphatidylinositol (GPI)-anchored C-terminus^16^. Tetherin is localized in lipid rafts at the cell surface and on intracellular membranes^16^. Tetherin inhibits the release of not only HIV-1 but also that of a wide variety of enveloped viruses including other retroviruses, herpesviruses, filoviruses, and arenaviruses^17^,^18^,^19^. Several lentiviral proteins have acquired the ability to antagonize the antiviral activity of tetherin; these include Vpu, Env, and Nef in the case of HIV-1, HIV-2, and SIV, respectively. Several mechanisms have been proposed for the Vpu-mediated downregulation of tetherin. Vpu (i) removes tetherin from sites of virus budding, (ii) enhances degradation of tetherin, and (iii) down-regulates cell surface tetherin expression. The down regulation of cell surface tetherin by Vpu is in part due to slowing down the plasma membrane access of newly synthesized tetherin by trapping within the Golgi network. Vpu-induced downregulation of tetherin cell-surface expression is also associated with a ubiquitin-dependent lysosomal degradation through the ESCRT machinery that involves the recruitment of the β-TRCP E3 ubiquitin ligase (reviewed in^20^,^21^).

The small glutamine-rich tetratricopeptide repeat (TPR)-containing protein α (SGTA) contains three TPR domains, a 34-amino acid structural motif consisting of eight loosely conserved amino acid residues that form antiparallel α-helical hairpins and serve as scaffolds to mediate protein-protein interactions. SGTA is a ubiquitously expressed co-chaperone that binds directly to Hsp70 and Hsp90^22^. SGTA also interacts with the androgen^23^,^24^, glucocorticoid^24^ and progesterone receptors^24^, and negatively regulates their activity. Knockdown or depletion of SGTA enhances receptor activity, whereas its overexpression suppresses receptor function^24^. SGTA was identified as a cellular binding partner for several viral proteins: HIV-1 Vpu^25^,^26^,^27^, severe acute respiratory syndrome coronavirus protein 7a^28^, Rec protein of human endogenous retrovirus K^29^, and NS-1 of parvovirus H-1^30^. The TPR domain of SGTA was shown to interact with these viral accessory proteins. SGTA is reportedly involved in several cellular functions including cell division, apoptosis, intracellular transport, molecular co-chaperoning, and virus assembly and release. Accumulating evidence suggests the involvement of SGTA in a variety of cancers (reviewed in^31^,^32^).

Recent reports demonstrate a crucial role for SGTA in quality control of misfolded ER proteins or mislocalized membrane proteins. SGTA contains a noncanonical ubiquitin-like binding domain that interacts with Ubl4b, a component of the Bag6 complex. This interaction recruits SGTA to the Bag6 complex. It was suggested that the interaction of SGTA with Bag6 is required to promote the degradation of mislocalized proteins by ERAD^33^. In contrast, studies from High’s lab showed that for degradation of mislocalized membrane proteins, SGTA competes with the Bag6 complex for cytosolic mislocalized membrane proteins (MLPs) and prevents the ubiquitylation of specific precursors^34^. When SGTA is overexpressed, the efficient removal of MLPs by proteasomal degradation is delayed, resulting in their increased steady-state levels and cytosolic aggregation^34^. In another report, Leznicki *et al.* showed that overexpressed SGTA binding to Rpn13, an intrinsic ubiquitin receptor of the 26S proteasome regulatory subunit, resulted in an increase in steady-state levels of MLPs due to escape from proteasomal degradation^35^. It has been reported that SGTA and its yeast homolog Sgt2 promote the insertion of tail-anchored proteins into the ER membrane by direct interaction with a hydrophobic substrate^36^.

SGTA was first identified as a Vpu-interacting protein^25^. Overexpressed SGTA was reported to interact with HIV-1 Gag in a manner that could be reversed by Vpu. It was proposed that in the absence of Vpu, SGTA interacts with HIV-1 Gag and forms a complex that is inhibitory to HIV-1 release. In the presence of Vpu, due to stable interactions between SGTA and Vpu, the SGTA-Gag interactions are abrogated and the defect imposed by SGTA overexpression is reversed. Dutta and Tan further reported that the TPR domain of SGTA is sufficient for the inhibition of HIV-1 particle release^27^.

In this study, we performed a yeast two-hybrid screen with HIV-1 Vpu as bait with the aim of identifying Vpu-interacting proteins to better understand Vpu function. One of the proteins identified in this screen was SGTA. Because previous work on SGTA did not evaluate the effect of SGTA depletion on HIV-1 assembly and release, and did not examine a possible connection between Vpu, SGTA, and tetherin, we investigated the effect of SGTA depletion and overexpression on HIV-1 particle production in the presence and absence of Vpu and tetherin. We confirmed that SGTA overexpression impairs HIV-1 release; however, this phenomenon was independent of Vpu and tetherin expression. While we observed that SGTA depletion did not significantly impact virus release or Vpu-mediated tetherin degradation, we demonstrated that SGTA overexpression induced a marked stabilization and cytosolic accumulation of a non-glycosylated, 23-kDa form of tetherin. We propose that the accumulation of 23-kDa tetherin is not a result of inhibition of ERAD but likely due to a block in the translocation of tetherin across the ER membrane.

## Results

### Confirmation of SGTA as a Vpu-interacting protein by Y2H Screening

To identify cellular factors involved in Vpu function, we carried out yeast two-hybrid (Y2H) screening using full-length Vpu as bait (for details, see experimental procedures). We identified five Vpu-interacting proteins, including SGTA, which was previously reported to bind Vpu^25^. Callahan *et al.* previously observed that SGTA overexpression inhibited HIV-1 release, but the mechanism of action was not established. Also, this previous study was performed before tetherin was identified as the key cellular protein counteracted by Vpu to stimulate virus release. Thus, in this study, we investigated the role of SGTA in Vpu-mediated tetherin degradation and HIV-1 release.

### Knockdown of SGTA has no effect on HIV-1 release

**Figure 1:**
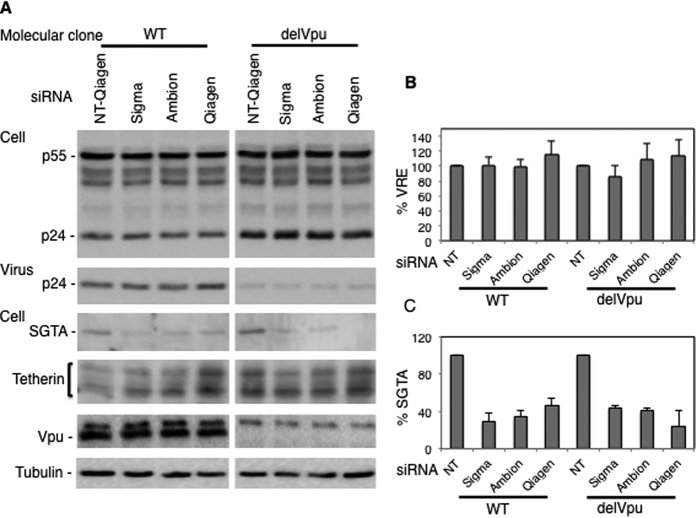
SGTA depletion has no effect on HIV-1 release. (**A**) HeLa cells were transfected with non-targeting siRNA (NT-Qiagen) or SGTA siRNA (from Sigma, Ambion, and Qiagen) and 4 h later were transfected with WT or Vpu-defective (delVpu) pNL4-3 HIV-1 molecular clones. siRNA transfection was repeated again the next day. One day later, cell and viral lysates were prepared and subjected to western blot analysis with HIV-Ig to detect the Gag precursor Pr55Gag (p55) and the CA protein p24, or antibodies specific for SGTA, tetherin, Vpu, or tubulin. (**B**) virus release efficiency (VRE) was calculated as the amount of virion-associated p24 (CA) relative to total Gag in cell and virus. VRE was set to 100% for NT siRNA. (**C**) relative expression of endogenous SGTA, NT siRNA was set to 100%. Data shown are ± SD from three independent experiments.

To investigate whether endogenous SGTA plays a role in HIV-1 release, we carried out knockdown experiments in HeLa cells. We used single or pooled siRNAs from three different sources (Sigma, Ambion, and Qiagen). An SGTA knockdown efficiency of up to 80% was achieved (Fig. 1A,C). Under these conditions, there was no significant effect on virus release for HIV-1 molecular clones either expressing (WT) or not expressing (delVpu) the HIV-1 accessory protein Vpu (Fig. 1A,B). The knockdown of SGTA also had little or no effect on the expression of tetherin and no obvious toxicity to the cells was observed as determined by unchanged levels of tubulin expression (Fig. 1A).

### Overexpression of SGTA inhibits HIV-1 release in a Vpu-independent fashion

**Figure 2:**
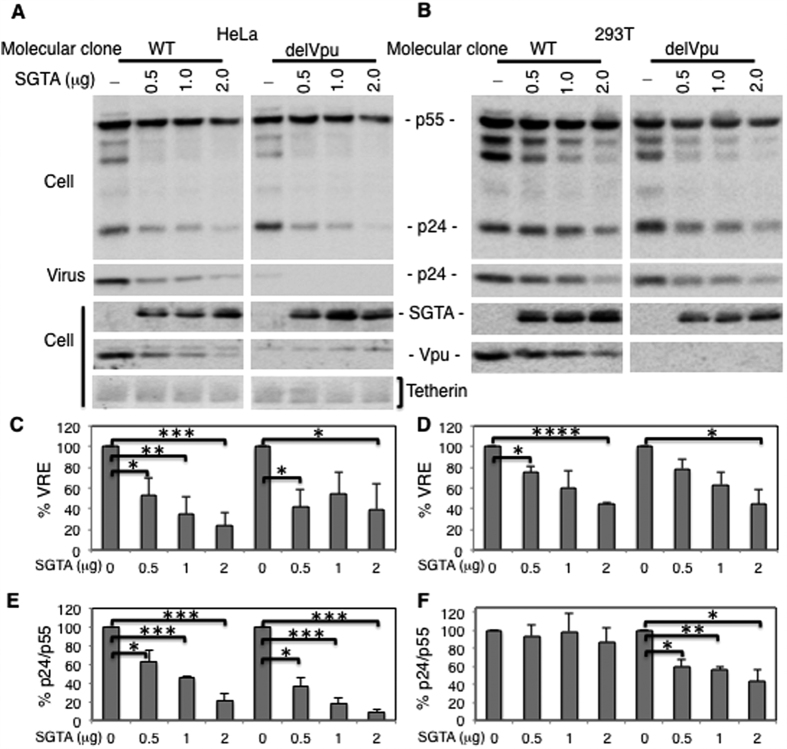
SGTA overexpression inhibits HIV-1 release. HeLa (**A**) and 293T (**B**) cells were transfected with WT or Vpu-defective (delVpu) pNL4-3 HIV-1 molecular clones with or without FLAG-tagged SGTA expression vector. One day posttransfection, cell and viral lysates were prepared and subjected to western blot analysis with HIV-Ig to detect the Gag precursor Pr55Gag (p55) and the CA protein p24, or anti- FLAG to detect FLAG-tagged SGTA, or anti-tetherin, or anti-Vpu antisera. (**C**,**D**) virus release efficiency (VRE) was calculated as in Fig. 1B, and VRE was set to 100% for no SGTA controls. (**E**,**F**) the ratio of p24 to p55 in cell lysates was calculated and set to 100% for no SGTA controls. Data shown are ± SD from three (**D**,**F**) or four (**C,E**) independent experiments. P values (two-tailed paired t-test): *p < 0.05, **p < 0.01, ***p < 0.005, ****p < 0.0005.

To investigate the effect of SGTA overexpression on HIV-1 particle release, we cotransfected cells with a vector expressing FLAG-tagged SGTA along with molecular clones encoding WT or Vpu-defective HIV-1. One day posttransfection, virus was concentrated, and cell and viral lysates were subjected to western blot analysis. As shown previously^25^,^27^, overexpression of SGTA reduced WT HIV-1 particle production over four-fold and that of delVpu HIV-1 two-fold in HeLa cells (Fig. 2A,C). This reduction in virus release in HeLa cells was accompanied by an approximately two-fold reduction in the total levels of cellular Gag (data not shown), and a five-fold reduction in Gag processing, as measured by the relative ratio of p24 (CA) to Pr55Gag (p55), for both WT and delVpu HIV-1 (Fig. 2A,E). In 293T cells, overexpression of SGTA reduced the release of both WT and delVpu HIV-1 by about two-fold (Fig. 2B,D). The defects in Gag processing in 293T cells were minimal with WT HIV-1; however, a two-fold reduction in the ratio of p24 to p55 was observed with delVpu HIV-1 (Fig. 2B,F). Total levels of cell-associated Gag were reduced by ~40% in 293T cells at the highest SGTA DNA input (data not shown). The extent of reduction in virus release imposed by SGTA overexpression was similar for WT and delVpu, and was also similar in HeLa cells, which express tetherin, and 293T cells, which do not. These results indicate that the ability of SGTA overexpression to inhibit HIV-1 particle production is not linked to Vpu or tetherin expression. To examine whether the inhibition of HIV-1 release resulted from the formation of a stable Gag-SGTA complex, we tested whether SGTA is incorporated into virions by probing released virions for SGTA. This analysis indicated that overexpressed, FLAG-tagged SGTA is not incorporated into virions at detectable levels (data not shown).

### Overexpression of SGTA in the presence of Vpu increases the levels of a 23-kDa form of tetherin

**Figure 3:**
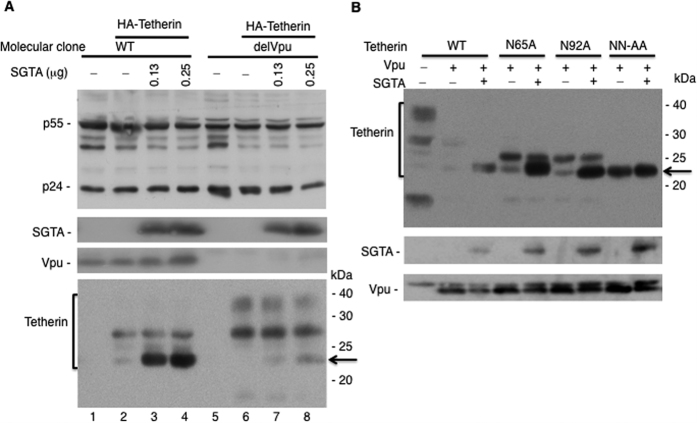
Overexpression of SGTA in the presence of Vpu increases the levels of a non-glycosylated 23-kDa form of tetherin. (**A**) 293T cells were transfected with WT or Vpu-defective (delVpu) pNL4-3 HIV-1 molecular clones, and vectors expressing HA-tagged human tetherin with or without FLAG-tagged SGTA expression vector. One day posttransfection, cell and viral lysates were prepared and subjected to western blot analysis with HIV-Ig to detect the Gag precursor Pr55Gag (p55) and the CA protein p24, anti-FLAG to detect FLAG-tagged SGTA or anti-HA to detect HA-tagged tetherin or anti-Vpu antisera. Mobility of molecular mass standards is indicated to the right of the anti-HA blot. The location of the 23-kDa tetherin species is indicated by the arrow. (**B**) 293T cells were transfected with vectors expressing HA-tagged WT or glycosylation-defective tetherin mutants (N65A, N92A and NN-AA) with or without FLAG-tagged SGTA and Vpu expression vectors. One day posttransfection, cells were lysed and subjected to western blot analysis with anti-HA to detect HA-tagged tetherin or anti-FLAG to detect FLAG-tagged SGTA or anti-Vpu antisera. Molecular mass markers are shown on the right of the anti-HA blot. The location of the non-glycosylated, 23-kDa tetherin species is indicated by the arrow.

During the course of our overexpression experiments designed to measure the effect of SGTA overexpression on virus release, we made the unexpected observation that SGTA overexpression in the context of WT HIV-1 led to a marked increase in the levels of a 23-kDa form of tetherin (Fig. 3A, lanes 3 and 4, bottom panel, arrow). This phenomenon required substantially less SGTA expression vector input (0.13 μg) relative to the amount needed to inhibit virus production (Fig. 2). The SGTA-mediated increase in levels of 23-kDa tetherin observed in the context of WT HIV-1 was much lower in the context of HIV-1 lacking Vpu (delVpu; Fig. 3A, compare lanes 3 and 4 with lanes 7 and 8). At these lower inputs of SGTA expression vector (0.13 and 0.25 μg), only a ~20% reduction in WT HIV-1 release was observed (data not shown). As expected^8^,^9^, in the absence of Vpu (Fig. 3A, lanes 6–8), high molecular weight tetherin bands were observed due to lack of Vpu-induced tetherin degradation. The results presented in Fig. 3A demonstrate that in the presence of Vpu, but not in its absence, SGTA overexpression markedly increases the abundance of a 23-kDa species of tetherin.

### The 23-kDa tetherin species is not glycosylated

Tetherin contains two major sites of N-linked glycosylation, residues Asn65 and Asn92^37^. To determine whether the 23-kDa tetherin species, whose abundance is increased by SGTA in the presence of Vpu, is glycosylated, we compared the expression pattern of WT and glycosylation-defective tetherin mutants. We expressed both single mutants (N65A and N92A) and a double mutant (NN-AA) in the absence and presence of SGTA and Vpu. Both single mutants showed a strong band at ~25 kDa in the absence of SGTA. In the presence of SGTA, the 23-kDa tetherin species was observed in both the single mutants as observed with the WT (Fig. 3B). The molecular mass of the non-glycosylated double mutant NN-AA is similar to that of the form of tetherin whose abundance is increased by SGTA in the presence of Vpu. These observations suggest that the 23-kDa form of tetherin that is apparently stabilized by SGTA is a non-glycosylated species.

### The C-terminus of SGTA is required for enhanced expression of the 23-kDa tetherin species

**Figure 4:**
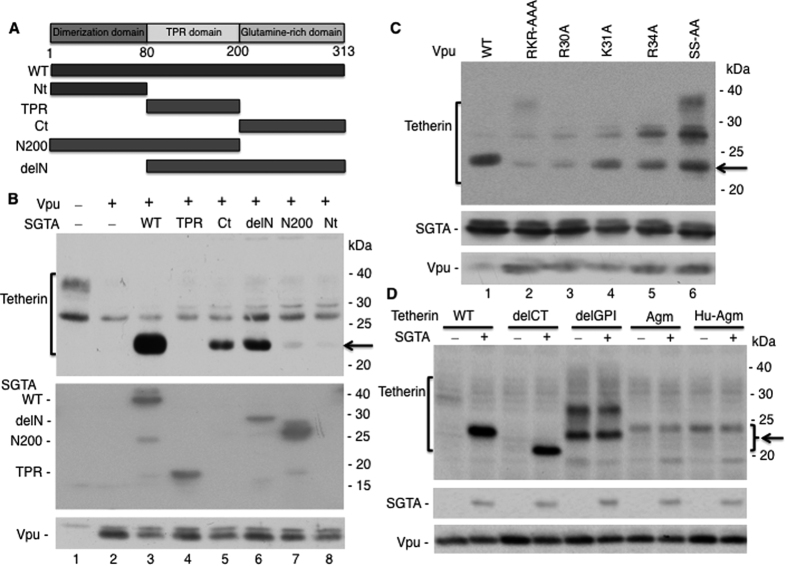
Domains of SGTA, Vpu and tetherin required for the SGTA-induced accumulation of non-glycosylated tetherin. (**A**) Truncated SGTAs that were tested to identify the domain of SGTA required for accumulation of non-glycosylated tetherin include the tetratricopeptide containing domain (TPR); the C-terminal domain (Ct); a mutant lacking the N-terminus (delN); the N-terminal domain (Nt); and the N-terminal plus TPR domains (N200). (**B**) 293T cells were transfected with the vector expressing HA-tagged tetherin with or without Vpu and FLAG-tagged SGTA expression vectors. One day posttransfection, cells were lysed and subjected to western blot analysis with anti-HA to detect HA-tagged tetherin or anti-FLAG to detect FLAG-tagged SGTA or anti-Vpu antisera. Mobility of molecular mass standards is shown on the right of anti-HA and anti-FLAG blots. The location of the non-glycosylated, 23-kDa tetherin species is indicated by the arrow. (**C**) 293T cells were transfected with vectors expressing FLAG-tagged SGTA, HA-tagged tetherin, and WT or mutant Vpu (RKR-AAA, R30A, K31A, R34A, and SS-AA). One day posttransfection, cells were lysed and subjected to western blot analysis with anti-HA antibodies to detect HA-tagged tetherin or anti-FLAG antibodies to detect FLAG-tagged SGTA or anti-Vpu antisera. Molecular mass markers are shown on the right of the anti-HA blot. The location of the non-glycosylated, 23-kDa tetherin species is indicated by the arrow. (**D**) 293T cells were transfected with vectors expressing HA-tagged tetherin and Vpu with or without FLAG-tagged SGTA expression vector. Tetherin variants tested to identify the regions of tetherin required for SGTA-mediated stabilization of non-glycosylated tetherin include a cytoplasmic tail deletion mutant (delCT); a GPI-anchor deletion mutant (delGPI); Agm tetherin (Agm); and a human tetherin containing the Agm transmembrane domain (Hu-Agm). One day posttransfection, cells were lysed and immunoblotted with anti-HA antibodies to detect HA-tagged tetherin or anti-FLAG antibodies to detect FLAG-tagged SGTA or anti-Vpu antisera. Mobility of molecular mass standards is shown on the right of anti-HA blot. The location of the non-glycosylated, 23-kDa tetherin species is indicated by the arrow.

SGTA contains an N-terminal dimerization domain that interacts with Ubl4A/Bag6, a central TPR domain that interacts with chaperones Hsp70 and Hsp90, and a glutamine-rich C-terminal domain that interacts with hydrophobic precursor proteins^33^,^38^,^39^ (Fig. 4A). To identify which domain of SGTA is required for increased levels of the non-glycosylated tetherin species, we used a panel of deletion mutants containing or lacking individual domains^33^ (Fig. 4A). 293T cells were co-transfected with FLAG-tagged WT SGTA or SGTA truncation mutants and tetherin and Vpu expression vectors. As demonstrated above, the 23-kDa tetherin species was highly expressed in the presence of WT SGTA (Fig. 4B, lane 3, arrow). While the SGTA TPR domain was expressed at a level similar to that of WT SGTA, the TPR domain alone did not increase the amount of 23-kDa tetherin (Fig. 4B, lane 4). Likewise, the N-terminal domain alone (Nt) or the N-terminal domain plus the TPR domain (N200) did not induce elevated levels of the non-glycosylated tetherin species. In contrast, overexpressing the C-terminal domain (Ct) alone or in combination with the TPR domain (delN) resulted in increased expression of the non-glycosylated tetherin species (Fig. 4B, lanes 5 and 6). As reported previously^33^, the Nt and Ct fragments were not detected by western blotting in cell lysates (Fig. 4B). To verify their expression, we immunostained SGTA with anti-FLAG antibodies and, as shown in Fig. S1, we could detect the expression of SGTA Nt and Ct domains at a level comparable to that of WT SGTA. From these results, we can conclude that the C-terminus of SGTA is necessary and sufficient for the SGTA-mediated increase in expression of the non-glycosylated tetherin species.

### Membrane-proximal basic residues of Vpu are required for the SGTA-mediated increase in non-glycosylated tetherin expression

Next, we asked which residues of Vpu are required for the SGTA-mediated increase in non-glycosylated tetherin levels. Vpu is an oligomeric type I integral membrane protein with a hydrophilic, highly basic domain projecting from the cytoplasmic membrane face^40^. This basic patch (Vpu residues R30, K31, and R34) has previously been implicated in Vpu binding to SGTA^27^. We mutated all three of these basic residues, either together (RKR-AAA) or individually (R30A, K31A, R34A). We co-expressed tetherin and SGTA in the presence of WT or mutant Vpu in 293T cells. As shown above, in the presence of SGTA and WT Vpu, high levels of non-glycosylated tetherin were detected (Fig. 4C, lane 1). In contrast, in the presence of the RKR Vpu mutant, the non-glycosylated tetherin species did not accumulate (Fig. 4C, lane 2). Of the three individual basic residue mutants, R30A was the most defective in its ability to increase non-glycosylated tetherin expression (Fig. 4C, lane 3). The triple mutant was also partially defective in its ability to degrade the glycosylated forms of tetherin, (~30–36 kDa) compared to the non-phosphorylated Vpu mutant (SS-AA)^3^ (Fig. 4C). However, unlike the RKR-AAA triple mutant, all single mutants down-regulate the highly glycosylated tetherin species (Fig. 4C). Because of its inability to degrade tetherin, the RKR-AAA triple and SS-AA mutants were unable to rescue virus release in the presence of tetherin (data not shown). The expression of all Vpu mutants was comparable to, or higher than, that of WT (Fig. 4C).

### The transmembrane domain of human tetherin is required for the SGTA-mediated increase in 23-kDa tetherin expression

Having identified the domains of SGTA and the residues of Vpu that contribute to the SGTA-mediated elevation in expression of non-glycosylated tetherin, we next wanted to identify which domain of tetherin is essential for this phenomenon. We constructed several tetherin mutants to identify the domain(s) required for this increased expression. As shown in Fig. 3B, both single non-glycosylation mutants (N65A, N92A) were stabilized by SGTA overexpression in the presence of Vpu. The non-dimerizing mutant (CCC)^37^, and ubiquitylation mutants (2STS, K17,20A, and STSKK)^41^ showed increased levels of the non-glycosylated tetherin species in the presence of Vpu and SGTA (Fig. S2) suggesting that neither dimerization nor ubiquitylation of tetherin is required for increased expression of 23-kDa tetherin induced by SGTA. Expression of the tetherin cytoplasmic tail deletion mutant (delCT) was increased by SGTA and Vpu (Fig. 4D) suggesting that the cytoplasmic tail is not required for SGTA-mediated stabilization of non-glycosylated tetherin. The GPI-anchor mutant (delGPI)^8^ expressed the non-glycosylated tetherin species even in the absence of SGTA (Fig. 4D). Further studies will be required to define the requirement of the GPI-anchor for SGTA-mediated stabilization of tetherin. We also tested two forms of tetherin that are not downregulated by Vpu due to a loss of Vpu-tetherin interaction: the tetherin from African green monkey (Agm) and a chimeric tetherin containing the Agm transmembrane domain in human tetherin (Hu-Agm)^11^. As shown in Fig. 4D, neither the Agm tetherin nor the Hu-Agm chimera was stabilized by SGTA. These results indicate that the transmembrane domain of human tetherin is essential for SGTA-mediated stabilization of the non-glycosylated tetherin species, most likely because this domain is critical for the tetherin-Vpu interaction.

### The non-glycosylated tetherin species co-immunoprecipitates with SGTA in the presence of WT Vpu but not in the presence of the membrane-proximal basic residue Vpu mutant

**Figure 5:**
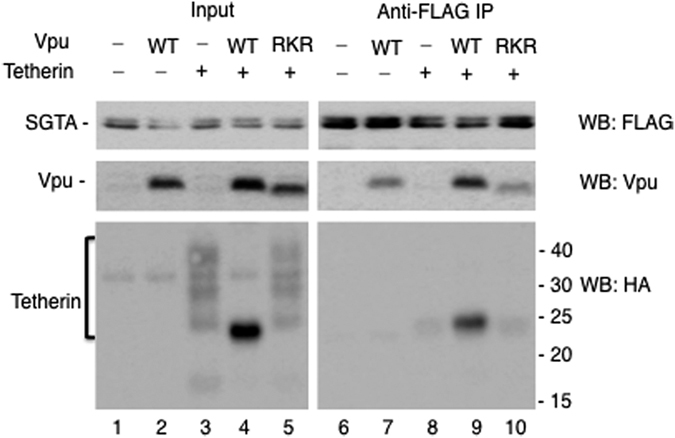
The non-glycosylated tetherin species co-immunoprecipitates with SGTA in the presence of WT Vpu. 293T cells were transfected with the vector expressing FLAG-tagged SGTA without or with Vpu (WT or RKR mutant) and HA-tagged tetherin expression vectors. Twenty-four h post-transfection, cells were lysed and immunoblotted with anti-FLAG (top left panel), anti-Vpu (middle left panel), or anti-HA antibodies (lower left panel) or immunoprecipitated with agarose beads conjugated with anti-FLAG antibodies followed by immunoblotting with anti-FLAG (top right panel), anti-Vpu (middle right panel) or anti-HA antibodies (lower right panel) to detect FLAG-tagged SGTA, Vpu and HA-tagged tetherin, respectively. The mobility of molecular mass standards is shown on the right.

The data presented in Fig. 4D suggest that the tetherin-Vpu interaction is required for the ability of SGTA to increase expression of the non-glycosylated species. Because SGTA is a Vpu-interacting protein, this raises the possibility that a ternary SGTA-Vpu-tetherin complex forms in cells expressing these three proteins. To test this hypothesis, we carried out co-immunoprecipitation assays. 293T cells were transfected with FLAG-tagged SGTA in the absence and presence of Vpu (WT and RKR mutant) and HA-tagged tetherin. As shown in Fig. 5, although several forms of tetherin are expressed in cells, only the 23-kDa tetherin species is co-immunoprecipitated with SGTA in the presence of Vpu. We also observed Vpu in the co-immnoprecipitate (Fig. 5). Compared to WT Vpu, only 60 ± 5.2% of the Vpu RKR mutant was co-immunoprecipitated by SGTA when normalized to input (average of three independent experiments). These results support the existence of a ternary SGTA-Vpu-tetherin interaction.

### The non-glycosylated tetherin species localizes to the cytosol

**Figure 6:**
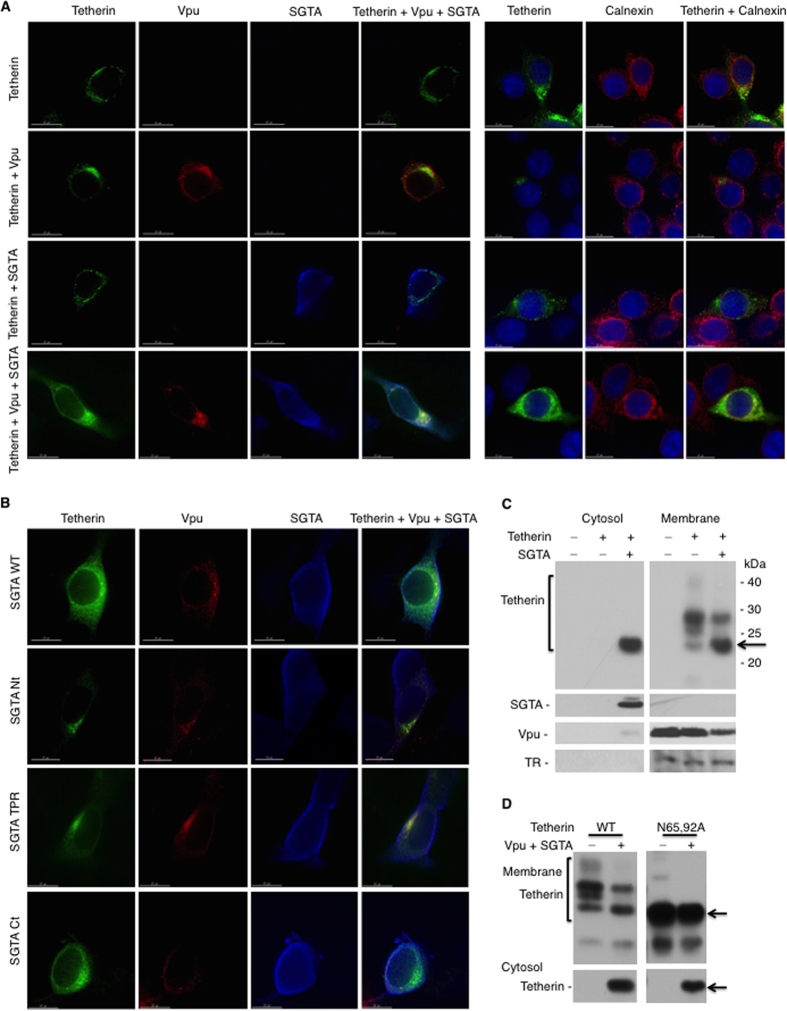
The non-glycosylated tetherin species partially localizes to the cytosol. (**A**,**B**) 293T cells were transfected with the vector expressing HA-tagged tetherin with or without Vpu and FLAG-tagged SGTA (WT and individual domains) expression vectors. (**A**) twenty-four h post-transfection, cells were fixed and co-stained with either anti-HA, anti-Vpu, and anti-FLAG, or anti-HA and anti-calnexin antibodies and images were acquired with a Delta-Vision deconvolution microscope. (**B**) cells were fixed after 24 h post-transfection and co-stained with anti-HA, anti-Vpu, and anti-FLAG antibodies. Scale bars in (**A,B**) are identical and represents 15 μm. (**C**) 293T cells were transfected with vectors expressing Vpu alone or with HA-tagged tetherin in the presence and absence of SGTA. Twenty-four h post-transfection cells were sonicated, and membrane and cytosolic fractions were separated by ultracentrifugation and immunoblotted with anti-HA antibodies to detect HA-tagged tetherin or anti-FLAG antibodies to detect FLAG-tagged SGTA or antibodies specific for Vpu or transferrin receptor. Molecular mass markers are shown on the right of the anti-HA blot. (**D**) 293T cells were transfected with vectors expressing WT tetherin or the non-glycosylated (NN-AA) tetherin mutant with or without Vpu and FLAG-tagged SGTA expression vectors. The cytosol and membranes were fractioned as in (**C**) and immunoblotted with anti-HA antibodies to detect HA-tagged tetherin. The location of the non-glycosylated, 23-kDa tetherin species is indicated by the arrow.

Tetherin is a type II membrane protein that contains two membrane anchors, a transmembrane domain at the N-terminus, and a GPI-anchor at the C-terminus^16^. Because of the two membrane anchors, tetherin is highly membrane-associated, specifically in cholesterol- and sphingolipid-rich membrane microdomains^16^. To better understand the mechanism by which SGTA and Vpu expression increases the levels of 23-kDa tetherin, we performed immunofluorescence and membrane fractionation experiments. HA-tetherin was expressed in 293T cells in either the absence or presence of Vpu and SGTA, and cells were fixed and stained with anti-HA antibody. As shown in Fig. 6A, human tetherin is localized both on the cell surface and in intracellular compartments. As reported^9^, when Vpu is co-expressed with tetherin, the cell surface expression of tetherin appears to be reduced due to Vpu-mediated down-regulation. As shown earlier^42^, tetherin and Vpu colocalize in the intracellular compartments (Fig. 6A). Upon SGTA overexpression, the localization pattern of tetherin is not altered. However, in the presence of both Vpu and SGTA, the tetherin localization pattern shifts markedly to cytosolic and partially ER (as indicated by colocalization with the ER marker calnexin) (Fig. 6A right panel). Further, we overexpressed individual SGTA domains and examined localization of tetherin in the presence of Vpu. As shown in Fig. 6B, in the presence of WT SGTA or SGTA Ct, the cytosolic staining of tetherin increased, whereas in the presence of Nt or TPR domains of SGTA tetherin colocalized with Vpu in the intracellular compartments as observed in the absence of SGTA (Fig. 6A, left panel, second row). These results suggest that the non-glycosylated tetherin species is partially mislocalized to the cytosol in the presence of Vpu and SGTA, and that the C-terminal domain of SGTA is critical for this relocalization.

To extend this microscopy-based localization analysis, we performed biochemical fractionation studies. 293T cells expressing tetherin alone or coexpressing tetherin and Vpu and SGTA were sonicated and separated into cytosolic and membrane fractions. As expected, in the absence of SGTA, tetherin fractionated to membranes (Fig. 6C, lanes 2 and 5). However, in the presence of SGTA, the non-glycosylated tetherin species became highly expressed and ~50% fractionated to the cytosol (Fig. 6C, lanes 3 and 6). These biochemical results support the increased cytosolic localization of tetherin observed in the microscopy analysis and suggest that in the presence of SGTA and Vpu tetherin partially redistributes to the cytosol. Interestingly, in the absence of SGTA, Vpu fractionates to membranes (Fig. 6C, lanes 4 and 5), but in the presence of SGTA it partially fractionates to the cytosol (Fig. 6C, lane 3). The integral membrane protein transferrin receptor (TR) was present exclusively in membrane fractions (Fig. 6C), demonstrating that cytosolic fractions were not contaminated with membrane-associated material. As reported^26^, SGTA was present exclusively in the cytosolic fractions (Fig. 6C, lane 3). We also tested the impact of SGTA overexpression on cytosolic localization of the non-glycosylated tetherin mutant (NN-AA). As shown above, in the presence of SGTA, the WT 23-kDa tetherin species partially fractionates to the cytosol (Fig. 6D, lanes 1 and 2). Interestingly, although SGTA and Vpu coexpression does not increase the levels of the 23-kDa NN-AA mutant (Figs 3B and 6D), coexpression of SGTA and Vpu results in a significant increase in the levels of the non-glycosylated mutant in the cytosol (Fig. 6D, lanes 3 and 4). This further confirms that the cytosolic localization of the non-glycosylated tetherin species is SGTA-dependent.

### SGTA overexpression does not interfere with Vpu-mediated degradation of CD4

To determine the effects of SGTA overexpression on the behavior of another cellular protein downregulated by Vpu, we examined the effect of SGTA overexpression on CD4 levels. It is well established that the plasma membrane protein CD4 is downregulated by Vpu at least in part through a proteasomal degradation pathway^7^, more specifically by ERAD^43^. We expressed CD4 in 293T cells in the absence and presence of Vpu and SGTA. Unlike tetherin, CD4 was degraded by Vpu to a similar extent in the presence and absence of overexpressed SGTA (Fig. S3). This result also suggests that overexpression of SGTA does not interfere with ERAD, at least for the substrates tested.

### Accumulation of the non-glycosylated tetherin species is not due to inhibition of ERAD

**Figure 7:**
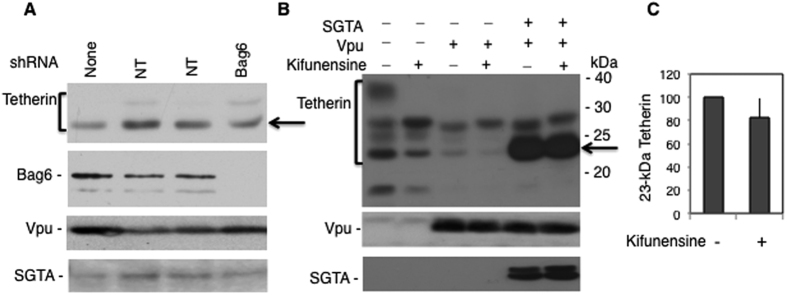
Inhibition of ER-associated degradation has no effect on the accumulation of the non-glycosylated tetherin species. (**A**) 293T cells were not transfected (none) or transfected with non-targeting (NT) or Bag6 shRNA constructs. One day later, the shRNA transfection was repeated. The cells were then transfected with vectors expressing HA-tagged tetherin, FLAG-tagged SGTA and Vpu. One day later, cells were lysed and subjected to western blotting with anti-HA, anti-Bag6, anti-Vpu, and anti-FLAG antibodies. (**B**) 293T cells were transfected with the vector expressing HA-tagged tetherin with or without Vpu and FLAG-tagged SGTA expression vectors. Eight h post-transfection, cells were treated or not treated with kifunensine for 16–18 h and cells were lysed. Cell lysates were subjected to immunoblotting with anti-HA antibodies to detect HA-tagged tetherin, anti-FLAG antibodies to detect FLAG-tagged SGTA or anti-Vpu antisera. The location of the non-glycosylated, 23-kDa tetherin species is indicated by the arrow. (**C**) the intensity of non-glycosylated, 23-kDa tetherin species in lanes 5 and 6 was quantified from four independent experiments.

It has been reported that Vpu induces the ERAD of tetherin^44^. We tested if the accumulation of the non-glycosylated tetherin species in the presence of SGTA is due to inhibition of the ERAD pathway. We used shRNA to deplete Bag6, a central component of the ERAD complex^45^. As shown in Fig. 7A, Bag6 depletion is highly efficient. In Bag6-depleted cells, the 23-kDa tetherin species is observed at levels similar to those in cells transfected with non-target shRNA. Thus, we can conclude that Bag6 is not required for SGTA-mediated stabilization of non-glycosylated tetherin. We also inhibited the ERAD pathway by treating cells with kifunensine, an ERAD inhibitor^46^. Kifunensine is an alkaloid compound that suppresses ER-associated mannosidase I activity^47^. Tetherin is modified with high-mannose oligosaccharide side chains in the ER; these are trimmed by the mannosidase I enzyme and modified to complex side chains in the Golgi^48^. Mannose trimming from the N-glycans is also essential for targeting misfolded glycoproteins for ERAD^49^. In the presence of kifunensine, there is a reduction in highly glycosylated tetherin, indicating that the compound is active. However, kifunensine treatment did not increase the levels of the 23-kDa tetherin species. Further, kifunensine had no effect on the SGTA-mediated stabilization of non-glycosylated tetherin (Fig. 7B, lanes 5 and 6 and Fig. 7C), suggesting that accumulation of the non-glycosylated tetherin species is not a result of inhibition of ERAD by SGTA overexpression.

### In the presence of Vpu, SGTA overexpression stabilizes non-glycosylated tetherin

**Figure 8:**
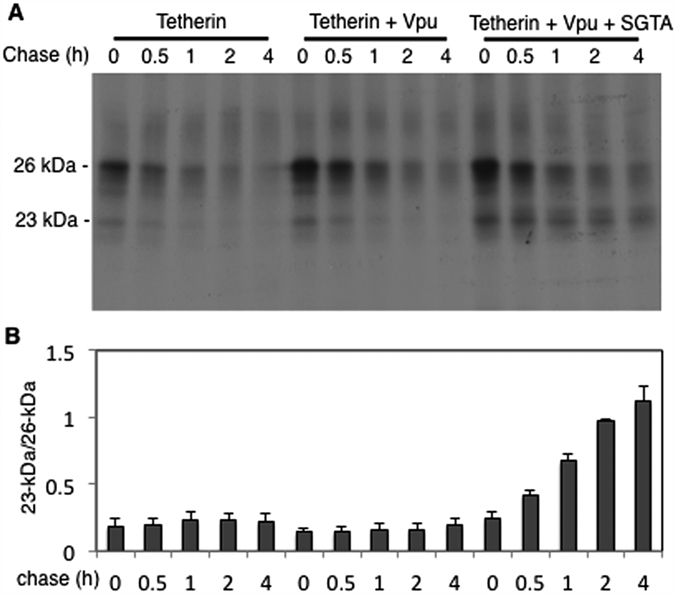
SGTA overexpression stabilizes non-glycosylated tetherin in the presence of Vpu. (**A**) 293T cells were transfected with the vector expressing HA-tagged tetherin with or without Vpu and FLAG-tagged SGTA expression vectors. 24 h post-transfection, cells were labeled with [^35^S]Met/Cys for 30 min and then chased for 0, 0.5, 1, 2, or 4 h. Cells were lysed and immunoprecipitated with anti-HA antibodies and analyzed by SDS-PAGE followed by fluorography. Positions of 26-kDa (glycosylated) and 23-kDa (non-glycosylated) tetherin are indicated. (**B**) the ratio of 23- to 26-kDa tetherin species was calculated from three independent experiments, ± SD. This ratio increased significantly over time in the presence of SGTA and Vpu, p < 0.01.

To elucidate the mechanism by which SGTA increases expression of the non-glycosylated tetherin species, we performed pulse-chase analysis. 293T cells expressing tetherin alone or coexpressing tetherin with Vpu or Vpu + SGTA were pulse-labeled for 30 min and chased for 0, 0.5, 1, 2, 4 h in unlabeled media. In the absence of SGTA, both 23-kDa and 26-kDa species of tetherin underwent degradation; however, in the presence of SGTA and Vpu, the degradation of the 23-kDa tetherin species was much less rapid (Fig. 8). As a result, we observed a striking increase in the ratio of 23- to 26-kDa forms of tetherin over time in cells overexpressing SGTA and Vpu. In contrast, the ratio of 23- to 26-kDa forms of tetherin was stable in cells expressing tetherin alone or tetherin plus Vpu. These results indicate that in the presence of Vpu, SGTA overexpression prevents the degradation of non-glycosylated tetherin leading to its stabilization and time-dependent accumulation.

## Discussion

In our Y2H screen, we identified SGTA as a Vpu-binding protein, consistent with a previous report^25^. Earlier studies showed that overexpression of SGTA disrupted HIV-1 release^25^,^27^, raising the possibility that SGTA overexpression abrogated Vpu-mediated tetherin antagonism. Because these earlier studies preceded the discovery of tetherin as the primary target of Vpu in enhancing virus particle release, we revisited this issue here. While we confirmed that SGTA overexpression did indeed interfere with HIV-1 release, several observations suggest that this phenomenon is not due to an effect on Vpu-mediated tetherin degradation: 1) SGTA overexpression inhibits the release of both Vpu(+) and Vpu(−) virus, 2) the effect of SGTA overexpression was seen in both tetherin-positive (HeLa) and tetherin-negative (293T) cells, and 3) the inhibition of virus release imposed by SGTA overexpression is accompanied by a defect in Pr55Gag processing to CA, which is not a phenotype associated with loss of Vpu expression in tetherin-expressing cells^8^,^50^. As suggested in earlier studies, it is possible that SGTA could directly bind to HIV-1 Gag and form a complex that inhibits virus assembly^25^,^27^. In contrast to SGTA overexpression, knockdown of SGTA expression by ~three-fold has no significant effect on HIV-1 release in either HeLa or 293T cells, indicating that endogenous levels of SGTA are not sufficient to interfere with Gag assembly. These results also suggest that endogenous SGTA, at least in the cell types tested, does not significantly affect tetherin expression. At this time, we cannot exclude the possibility that our SGTA depletions were not sufficiently complete to elicit significant effects on virus assembly and release or on levels of tetherin expression. More complete knockdown of SGTA is poorly tolerated, making complete knockdown difficult to achieve^51^. Further studies will be required to pinpoint which step(s) in the particle assembly pathway are inhibited by SGTA overexpression.

During the course of the above-described experiments, we made the serendipitous observation that SGTA overexpression led to a marked accumulation of a 23-kDa tetherin species. The size of this product is consistent with that of non-glycosylated tetherin, and indeed a tetherin mutant bearing substitutions at the two reported sites of N-linked glycosylation^37^ co-migrated with the 23-kDa form. The SGTA-mediated accumulation appears to be specific for tetherin, as CD4, another host protein that is degraded by HIV-1 Vpu^5^,^6^,^7^, is unaffected by SGTA overexpression. We identified domains/residues of SGTA, Vpu and tetherin required for the SGTA-mediated accumulation of the non-glycosylated tetherin species. The glutamine-rich C-terminus of SGTA, membrane-proximal basic residues of Vpu, and the transmembrane domain of tetherin are essential for the accumulation of non-glycosylated tetherin. The partially cytosolic localization of 23-kDa tetherin, SGTA and Vpu suggests the formation of a ternary SGTA-Vpu-tetherin complex. Indeed, our co-immunoprecipitation experiments show that only non-glycosylated, 23-kDa tetherin, but not the higher-molecular-weight glycosylated forms, co-immunoprecipitated with SGTA in the presence of Vpu.

Because SGTA has been shown to be a component of the ERAD complex^33^, we initially hypothesized that overexpression of SGTA could disrupt the ERAD process, leading to accumulation of a misfolded, non-glycosylated tetherin species. To test this hypothesis, we disrupted ERAD by depleting Bag6, a central component of the ERAD complex^45^, or treated cells with kifunensin, an inhibitor of this degradation pathway^46^. Neither Bag6 depletion nor kifunensin treatment had any effect on the accumulation of 23-kDa tetherin. These results suggest that the accumulation of the non-glycosylated tetherin species is not due to inhibition of the ERAD pathway *per se*. To track the fate of newly synthesized tetherin in more detail, we performed pulse-chase analysis. In the absence of SGTA overexpression, we observed that both 26-kDa (glycosylated) and 23-kDa (non-glycosylated) tetherin undergo degradation with similar kinetics, leading to a ratio of 26-kDa to 23-kDa forms that remains constant over time. However, SGTA overexpression in the presence of Vpu (but not in its absence) resulted in the much-reduced degradation of the 23-kDa form of tetherin and a time-dependent increase in the ratio of 23- to 26-kDa tetherin species. Together, these findings suggest that overexpression of SGTA in the presence of Vpu leads to the formation of a ternary complex that results in subsequent accumulation of non-glycosylated tetherin in the cytosol.

Accumulating evidence in the literature suggests a role for SGTA in stabilization and cytosolic aggregation of MLPs. The Bag6 complex and SGTA play a key role in determining the fate of MLPs^34^,^52^,^53^,^54^. The Bag6 complex promotes the polyubiquitylation of MLPs followed by degradation via the proteasome pathway^33^,^53^, whereas SGTA was shown to antagonize this process by deubiquitylating MLPs and delaying their degradation, resulting in their accumulation and aggregation in the cytosol^34^,^52^. In a recent report, Leznicki *et al.* showed that SGTA interacts with Rpn13, the intrinsic proteasomal ubiquitin receptor, and that overexpression of SGTA resulted in an increase in the association of SGTA and its MLP substrate with the proteasome and, consequently, in an increase in MLP levels. The ability of SGTA overexpression to prevent MLP degradation in the proteasome was disrupted by either overexpressing the C-terminal, SGTA-binding region of Rpn13, or by mutating the TPR domain of SGTA, which interacts with Rpn13^35^. Overexpression of Bag6 also delayed the process of substrate degradation in a dominant-negative manner^55^. A single polyubiquitin chain can be captured by both Rpn10 and Rpn13 ubiquitin receptors^56^,^57^; based on these observations, Leznicki *et al.* proposed that Rpn10-bound Bag6 and Rpn13-bound SGTA can modulate the access of MLPs to the proteasome^35^. Our pulse-chase analysis indicates that the stabilization of 23-kDa tetherin by exogenous SGTA is likely due to a delay in the degradation of SGTA-bound 23-kDa tetherin. Our results add significant new information to these previous studies by showing that the C-terminal domain of SGTA, rather than the TPR domain, can elicit overexpression-induced increases in substrate stability. Our findings also demonstrate a requirement for a viral factor – HIV-1 Vpu – in the regulation of tetherin stability by SGTA. Several recent studies have shown that SGTA expression is upregulated in various types of cancer (reviewed in^31^,^32^), although it is not clear whether the increased expression of SGTA is the cause, or a consequence, of the pathogenic process. These observations suggest that there may be physiologically relevant situations (e.g., in cancer) under which SGTA overexpression alters the regulation of SGTA client proteins in a manner similar to that observed in this study. This question warrants further study. Although the physiological relevance of SGTA overexpression in HIV-1 biology is not clear, the inhibition of HIV-1 release induced by SGTA overexpression was observed independent of Vpu and tetherin, and was accompanied by a severe defect in Gag processing. Further studies will provide additional insights into the relationship between these viral and host cell factors and the cell biology of protein degradation and ER translocation.

## Methods

### Plasmids, antibodies, and chemicals

The full-length, infectious HIV-1 molecular clone pNL4-3^58^ and the Vpu-defective counterpart NL4-3delVpu^2^ have been described previously. The codon-optimized Vpu expression plasmid pcDNA-Vphu was used to express the Vpu^59^. pNL4-3delVpu and pcDNA-Vphu were kindly provided by K. Strebel. The N-terminally HA epitope-tagged tetherin expression vector, the deletion mutants of human tetherin delCT and delGPI, tetherin from African green monkey (agm), and chimeric human tetherin with agm transmembrane domain (Hu-Agm) were described previously^8^,^11^, and were generously provided by P. Bieniasz. PCR-based mutagenesis was used to introduce Ala substitutions at three cysteines (C53, C63, C91; referred to as CCC) and two asparagines (N65, N92), and serine, threonine and serine (S2,T3,S4 referred as 2STS), two lysines (K17, K20), and, in combination, serine, threonine and lysine (STSKK) residues in the cytoplasmic tail of tetherin. Anti-HA, anti-FLAG and anti-tubulin antibodies, anti-HA conjugated agarose beads, and kifunensine were purchased from Sigma (St. Louis, MO). Anti-human transferrin receptor antibody was purchased from Zymed Laboratories Inc. (San Francisco, CA), and anti-CD4 antibody was from Santa Cruz Biotechnology (Dallas, Texas). Anti-calnexin antibody was from Abcam (Cambridge, MA). Zenon antibody labeling kits and the Alexa Fluor 647 conjugated secondary antibody were from Invitrogen (Grand Island, NY). Anti-SGTA and anti-Bag6 antibodies were raised in rabbit^33^. Anti-Vpu^40^, anti-tetherin, and anti-HIV-1 Ig were obtained from the NIH AIDS Research and Reference Reagent Program.

### Yeast two-hybrid screening

The yeast two-hybrid screen was carried out at Myriad Genetics (Salt Lake City, UT). Briefly, the PNY200 yeast strain^60^ was co-transformed with linear pGBT.superB DNA^61^ and bait fragment to obtain recombinant plasmid to express Vpu fused to the GAL4 DNA binding protein (BD). The BK100 yeast strain^60^ was transformed with a prey cDNA library (isolated from brain, spleen, and macrophages), which expresses the various cDNA-encoded proteins as hybrids with the GAL4 transcriptional activation domain (AD). To perform the yeast two-hybrid screen, the PNY200 yeast strain expressing the GAL4-binding domain fusions was mated with the BK100 yeast strain. After mating, the cells are plated onto selective media, and if the bait and prey proteins interacted, transcription of two auxotrophic reporter genes (HIS3 and ADE2) with dissimilar promoters occurred^62^ and colonies were picked from the selection plates, and the prey inserts were identified by sequence analysis. To confirm interactions in this screen, the bait and prey plasmids were isolated from yeast diploids, and co-transformed into a native yeast strain, and a chemiluminescent reporter gene assay system^62^ was used to monitor the interaction.

### Cell culture and transfection

293T and HeLa cell lines were maintained in Dulbecco-modified Eagle’s medium (DMEM) containing 10% or 5% fetal bovine serum (FBS), respectively. One day after plating, the cells were transfected with appropriate plasmid DNA using Lipofectamine 2000 (Invitrogen Corp. Carlsbad, CA) according to the manufacturer’s recommendations. Cells and virus were harvested 24 h posttransfection and used for further analysis.

### Virus release assays

To knock down SGTA expression, we used siRNA from three different sources that were pools of four siRNAs (Sigma), three siRNAs (Ambion) or a single siRNA (Qiagen). One day after plating, HeLa cells were transfected with 100 nM non-target or SGTA siRNA with Oligofectamine transfection reagent (Invitrogen). Four hours later, cells were transfected with wild-type (WT) or Vpu-defective pNL4-3 molecular clones. The next day, medium was changed and the siRNA transfection was repeated. One-day later, virions were pelleted in an ultracentrifuge and cell and viral pellets were lysed^63^. In overexpression experiments, HeLa and 293T cells were co-transfected with pNL4-3 or pNL4-3delVpu in the absence or presence of tetherin and SGTA expression vectors. One-day posttransfection, virions were collected and cell and viral pellets were lysed as above. Viral proteins in cell and virus lysates were immunoblotted with HIV-Ig^63^ and virus release efficiency was calculated as the amount of virion-associated Gag as a fraction of total (cell- plus virion-associated) Gag.

### Western blotting analysis

For immunoblot analyses, cells were washed with PBS, and then lysed in a buffer containing 50 mM Tris-HCl (pH 7.4), 150 mM NaCl, 1 mM EDTA, 0.5% Triton X-100, and protease inhibitor cocktail (Roche life sciences, Basel, Switzerland). Proteins were denatured by boiling in sample buffer and subjected to SDS-PAGE, transferred to PVDF membrane, and incubated with appropriate antibodies as described in the text. Membranes were then incubated with HRP-conjugated secondary antibodies, and chemiluminescence signal was detected by using West Pico or West Femto Chemiluminescence Reagent (Thermoscientific, Waltham, MA). The protein bands were quantified by using Imagelab-Chemidoc (Bio-rad Laboratories, France).

### Immunoprecipitation of SGTA

To investigate the interactions between SGTA, Vpu and tetherin we carried out co-immunoprecipitation assays. 293T cells were transfected with FLAG-tagged SGTA in the absence and presence of vectors expressing human tetherin and Vpu (WT and RKR mutant). Twenty-four h post-transfection, cells were lysed in 0.5% IGEPAL and immunoprecipitations were performed with agarose beads conjugated with anti-FLAG antibodies. After a 12-h incubation, complexes were washed with 0.1% IGEPAL, and both cell lysates and immunoprecipitated samples were subjected to immunoblotting with anti-FLAG, anti-HA, and anti-Vpu antibodies.

### Immunofluorescence microscopy

For microscopy studies, 293T cells cultured in chamber slides were transfected with human tetherin in the absence or presence of Vpu and SGTA. Twenty-four h posttransfection, cells were rinsed with PBS and fixed with 3.7% paraformaldehyde in PBS for 30 min. The cells were rinsed with PBS, permeabilized with methanol at −20 °C for 4 min, washed in PBS and incubated with 0.1 M glycine-PBS for 10 min to quench the remaining aldehyde residues. After blocking with 3% BSA-PBS for 30 min, cells were incubated with Zenon Alexa Fluor 488-labeled anti-HA, and either Zenon Alexa Fluor 594-labeled anti-Vpu or Zenon Alexa Fluor 594-labeled anti-calnexin appropriately diluted in 3% BSA-PBS for 1 h. In triple antibody staining experiments, cells were first incubated with anti-FLAG antibody diluted in 3% BSA-PBS for 1 h, then cells were washed with PBS three times and then incubated with secondary antibody conjugated with Alexa Fluor 647 diluted in 3% BSA-PBS. After washing with PBS three times, cells were incubated with Zenon Alexa Fluor 488-labeled anti-HA and Zenon Alexa Fluor 594-labeled anti-Vpu. Finally, after washing with PBS three times, cells were mounted with Vectashield mounting media with DAPI (Vector Laboratories) and examined with a Delta-Vision RT microscope.

### Pulse-chase analysis

293T cells were transfected with HA-tagged human tetherin in the absence and presence of Vpu and SGTA expression vectors. One day posttransfection, cells were pulse-labeled with [^35^S]Met-Cys for 30 min and washed with DMEM containing 10% FBS and removed from the dish in the same medium. Cells were split into five equal parts and incubated for 0, 0.5, 1, 2, 4 h. Cells were spun down and lysed in 0.5% IGEPAL containing lysis buffer and immunoprecipitated with anti-HA antibodies and analyzed by SDS-PAGE followed by fluorography^64^.

## Additional Information

**How to cite this article**: Waheed, A. A. *et al.* The Vpu-interacting Protein SGTA Regulates Expression of a Non-glycosylated Tetherin Species. *Sci. Rep.* **6**, 24934; doi: 10.1038/srep24934 (2016).

## Supplementary Material

Supplementary Information
