# Supplementary material for: The Vpu-interacting Protein SGTA Regulates Expression of a Non-glycosylated Tetherin Species
Source: Sci Rep. 2016 Apr 22;6:24934. doi: 10.1038/srep24934 (PMC4840321; doi:10.1038/srep24934)
Supplement: Supplementary Information [file srep24934-s1.pdf]

The Vpu-interacting Protein SGTA Regulates Expression of a Non-glycosylated Tetherin Species

Abdul A. Waheed*[1], Scott MacDonald[1], Maisha Khan[1], Megan Mounts[1], Maya Swiderski[1], Yue Xu[2], Yihong Ye[2], and Eric O. Freed[1]

Supplementary Information

Supplementary Figure S1: WT, Nt, and Ct SGTA expression detectable by immunostaining.

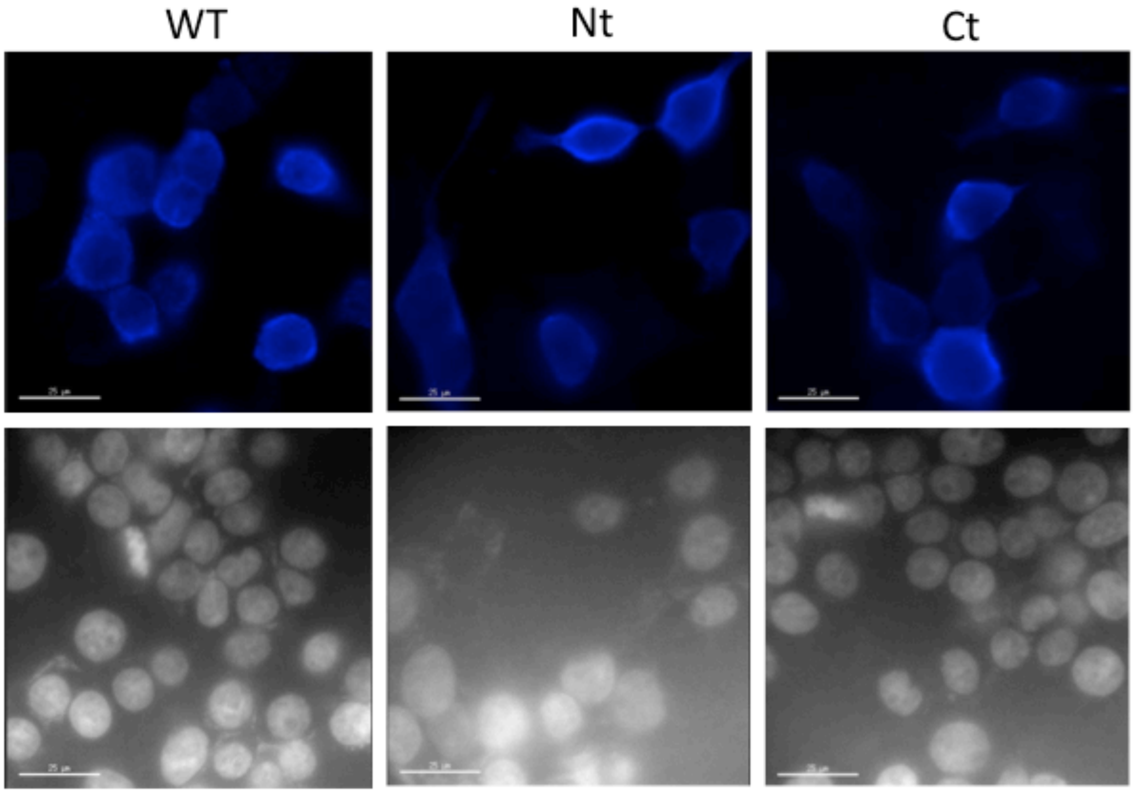

293T cells were transfected with FLAG-tagged SGTA (WT, Nt, and Ct) expression vectors. Twenty-four h post-transfection, cells were fixed and stained with anti-FLAG antibody followed by Alexa Fluor 647-conjugated secondary antibody, and images were acquired with a Delta-Vision deconvolution microscope. To compare the transfection efficiency, >50 FLAG-tag-expressing cells were counted from different fields and compared with the total number of cells in each field. The percentage of cells transfected were 26.2% for WT, 25.2% for Nt domain, and 21.8% for Ct domain of SGTA. Scale bars represent 15 μm.

Supplementary Figure S2. Dimerization and ubiquitylation of tetherin are not required for SGTA-mediated stabilization of non-glycosylated tetherin.

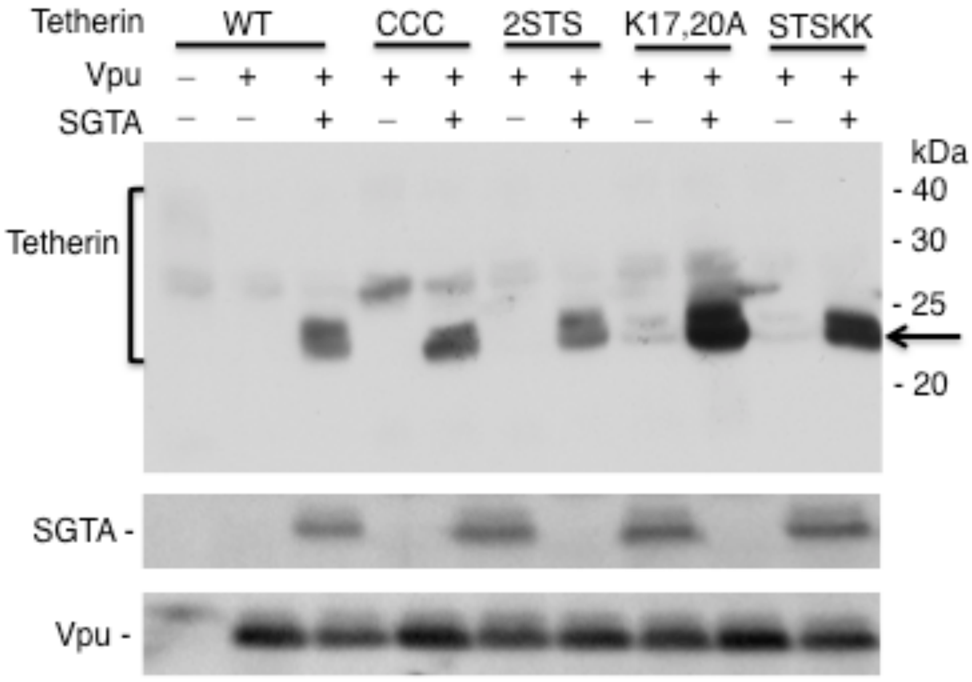

293T cells were transfected with vectors expressing HA-tagged WT tetherin or mutant tetherin (CCC - non-dimerizing mutant; 2STS, K17,20A, and STSKK- ubiquitylation site mutants) with or without Vpu and FLAG-tagged SGTA expression vectors. One day posttransfection, cells were lysed and subjected to western blot analysis with anti-HA antibodies to detect HA-tagged tetherin or anti-FLAG antibodies to detect FLAG-tagged SGTA or anti-Vpu antisera. Molecular mass markers are shown on the right of the anti-HA blot. The location of the non-glycosylated, 23-kDa tetherin species is indicated by the arrow.

Supplementary Figure S3. SGTA overexpression does not interfere with Vpu-mediated degradation of CD4.

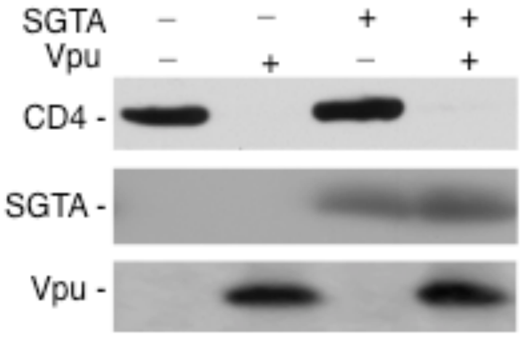

293T cells were transfected with a vector expressing CD4 with or without FLAG-tagged SGTA and Vpu expression vectors. One day posttransfection, cells were lysed and immunoblotted with anti-FLAG antibodies to detect FLAG-tagged SGTA or antibodies specific for CD4 or Vpu.
